# Accelerating massively parallel hemodynamic models of coarctation of the aorta using neural networks

**DOI:** 10.1038/s41598-020-66225-0

**Published:** 2020-06-11

**Authors:** Bradley Feiger, John Gounley, Dale Adler, Jane A. Leopold, Erik W. Draeger, Rafeed Chaudhury, Justin Ryan, Girish Pathangey, Kevin Winarta, David Frakes, Franziska Michor, Amanda Randles

**Affiliations:** 1 Department of Biomedical Engineering, Duke University, Durham, NC USA; 2 Lawrence Livermore National Laboratory, Livermore, CA USA; 3 Brigham and Women’s Hospital, Harvard Medical School, Boston, MA USA; 4 Department of Biological and Health Systems Engineering, Arizona State University, Tempe, AZ USA; 5 Department of Department of Data Sciences, Dana-Farber Cancer Institute, Boston, MA USA; 6 Department of Biostatistics, Harvard T. H. Chan School of Public Health, Boston, MA USA; 7 Department of Stem Cell and Regenerative Biology, Harvard University, Cambridge, MA USA; 8 The Broad Institute of Harvard and MIT, Cambridge, MA USA

**Keywords:** Computational models, Machine learning

## Abstract

Comorbidities such as anemia or hypertension and physiological factors related to exertion can influence a patient’s hemodynamics and increase the severity of many cardiovascular diseases. Observing and quantifying associations between these factors and hemodynamics can be difficult due to the multitude of co-existing conditions and blood flow parameters in real patient data. Machine learning-driven, physics-based simulations provide a means to understand how potentially correlated conditions may affect a particular patient. Here, we use a combination of machine learning and massively parallel computing to predict the effects of physiological factors on hemodynamics in patients with coarctation of the aorta. We first validated blood flow simulations against *in vitro* measurements in 3D-printed phantoms representing the patient’s vasculature. We then investigated the effects of varying the degree of stenosis, blood flow rate, and viscosity on two diagnostic metrics – pressure gradient across the stenosis (Δ*P*) and wall shear stress (WSS) - by performing the largest simulation study to date of coarctation of the aorta (over 70 million compute hours). Using machine learning models trained on data from the simulations and validated on two independent datasets, we developed a framework to identify the minimal training set required to build a predictive model on a per-patient basis. We then used this model to accurately predict Δ*P* (mean absolute error within 1.18 mmHg) and WSS (mean absolute error within 0.99 Pa) for patients with this disease.

## Introduction

Physiological factors that influence blood flow such as exercise, body temperature, and dehydration, and co-existing diseases, like anemia, polycythemia, and pyrexia can increase the severity of many cardiovascular diseases and the risk of morbidity and mortality^1–11^. Understanding the complex interplay between these conditions and how they affect both clinical and hemodynamic metrics is important in quantifying risks and improving patient care. Measuring interactions between physiological factors in a doctor’s office is difficult, and researchers and physicians are turning to hemodynamic simulations for risk stratification. Personalized blood flow simulations offer the ability to predict the effects of wide factor parameter spaces on hemodynamics, allowing patient-specific treatment plans that account for future risk^1,12^. Accurate hemodynamic simulations typically require extremely high computational demand due to complex geometries, unsteady flow profiles, and patient-adapted boundary conditions that lead to intricate and non-idealized flow patterns^13,14^. Recent advances in high performance computing have helped to decrease the time to solution for simulations^15,16^, but simulating all combinations of risk factors remains intractable. We therefore explored the use of machine learning (ML) models trained with simulations to predict such parameter spaces. By combining ML with principles from design of experiments, we developed a framework to identify and leverage the minimal simulation set required to build accurate predictive models on a per-patient basis, discerning how combinations of physiological factors impact key diagnostic metrics for patients with congenital heart disease.

In this work, we applied our novel framework to elucidate the interplay between physiological factors impacting blood flow parameters and clinically relevant outcomes in patients with coarctation of the aorta (CoA). CoA is one of the most prevalent congenital heart defects, affecting approximately 1,600 births each year in the U.S.^17^. CoA is characterized by a stenosis in the region distally adjacent to the left subclavian artery, creating a pressure gradient (Δ*P*) that can lead to cardiac failure, aortic and cerebral aneurysms, stroke, premature coronary artery disease, and hypertension^18^. A higher Δ*P* indicates increased patient risk and is used as a primary diagnostic metric to guide decisions regarding surgical intervention^19^. Studies simulating blood flow using computational fluid dynamics (CFD) have shown that certain physiological factors such as exercise can increase Δ*P* up to four-fold^1,12^ and that exercise and vessel geometry strongly influence local hemodynamics such as time-averaged wall shear stress (TAWSS), another important indicator of CoA severity. Although TAWSS is not used as a diagnostic measurement, evidence has shown that locations of abnormal TAWSS correlate with plaque build-up in the descending aorta (dAo) downstream of a stenosis^20–23^. However, a comprehensive evaluation of the influence of combinations of factors that affect blood flow in CoA has not been performed. Many of these factors are likely to impact TAWSS and Δ*P* by altering blood viscosity and flow rate, two parameters that influence hemodynamics throughout the vasculature. Thus, understanding and predicting the complex interplay between viscosity, flow rate, and important metrics such as TAWSS and Δ*P* is important for treating patients with CoA. However, the large number of potential combinations of physiological factors, manifested in viscosity-flow rate pairs, combined with the high computational cost of individual simulations, makes simulating all combinations impractical.

A recent approach to reduce computational overhead is to use ML models to predict the results of blood flow simulations. In these studies, large numbers of simulations were used to train and test an ML model, limiting simulations to one-dimensional (1D) or highly simplified 3D geometries. For example^24^, and^25^ trained neural networks with 1D simulations to predict how various coronary artery geometries influenced fractional flow reserve.^26^ went a step further and used a simplified 3D model consisting of a short straight vessel with a stenosis to train a neural network that predicted how the geometry impacted Δ*P*. While these straight vessel 3D and reduced order 1D geometries reduce run-time, they make limiting assumptions, increase uncertainty, and cannot capture complex local flow patterns such as blood recirculation, WSS, or vorticity^27,28^. Other recent ML models seek to predict underlying flow physics from measurements of passive scalars by directly incorporating fluid mechanics laws into the neural network cost functions^29^. These studies are a growing and interesting field, but they are generally confined to small domains and individual simulations. While we sought to predict clinically relevant simulation results across wide ranges of our parameter space with design of experiments, these previous works successfully demonstrate that neural networks can accurately predict the results of simulations.

**Figure 1:**
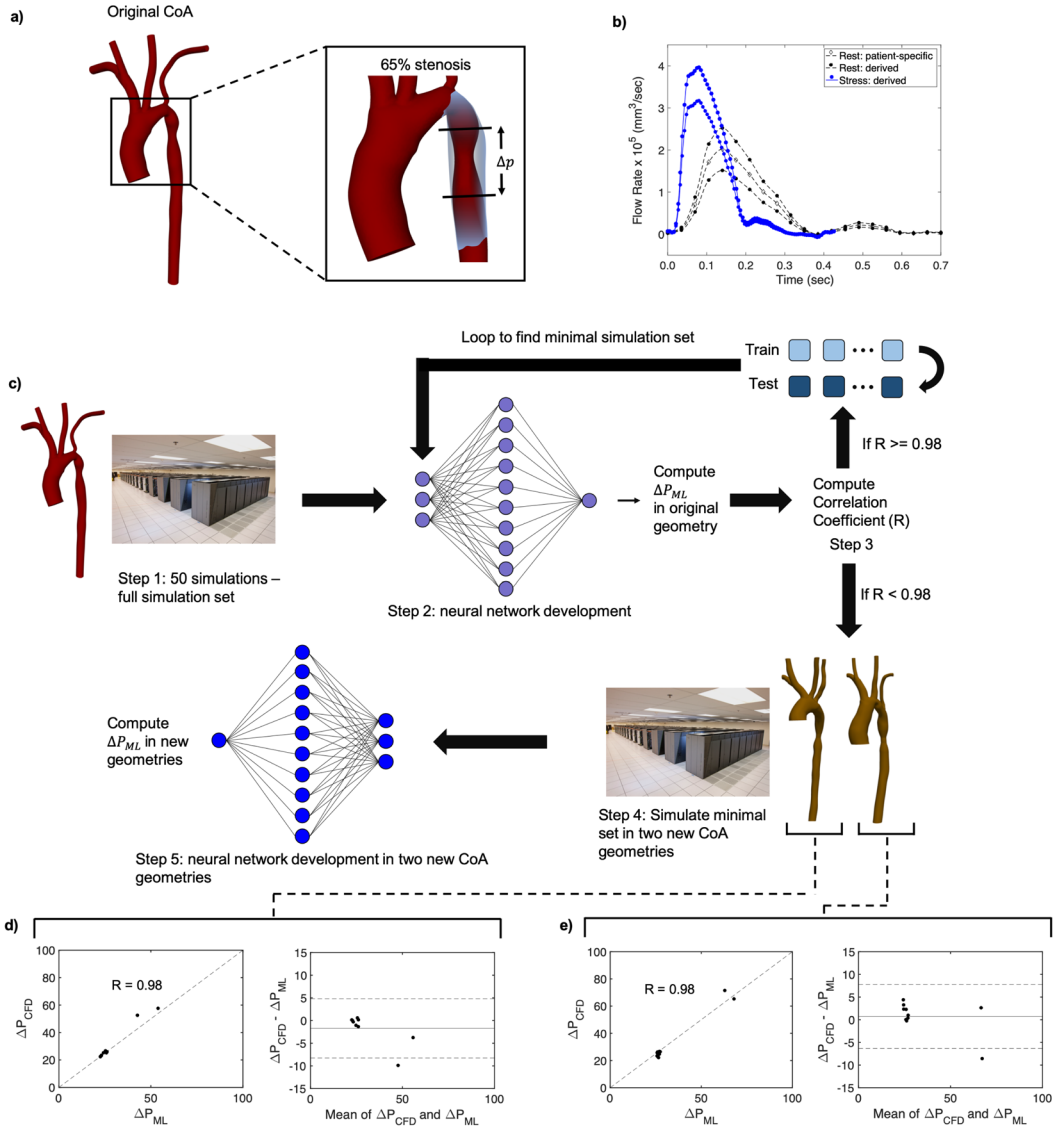
Design of experiments workflow to develop ML models. (**a**) 65% CoA geometry. (**b**) The five flow waveforms that were used as features representing a range between rest and exercise. (**c**) Design of experiments workflow. Step 1) Run 50 simulations in the patient-specific CoA (combination of ten viscosity values and five flow rates). Each simulation can be defined by a viscosity-flow rate pairing. Step 2) In the patient-specific CoA, train and test a neural network to predict Δ*P* from the simulations. Select 40 simulations for training and ten for the test set. Step 3) If the correlation coefficient R >= 0.98 in the test set, reduce the training set size by one, add that simulation to the test set, and return to Step 2. Otherwise, record the minimum simulation set defined by the viscosity-flow rate pairings in the training set, and return the correlation coefficient for analysis. Step 4) Test the robustness of the ML model by using the minimal simulation set defined by the viscosity-flow rate pairings to run simulations on two different CoA geometries. Also, run nine more simulations in the new geometries to use as a test set. Step 5) In the two new geometries, train and test neural networks to predict Δ*P* and compute the correlation coefficients in the test set. (**d**) ML model results for the 50 simulation set in the 65% CoA. (**e**) ML model results for one of the new 65% CoA geometries using nine simulations in the training set and nine simulations in the test set.

We hypothesized that a combination of design of experiments and ML could be used to minimize the computational burden of patient-specific simulations and provide a quantitative method to accurately predict hemodynamic risk factors across wide parameter spaces. To test this hypothesis, we developed a large-scale computational framework that uses design of experiments principles to drive massively parallel fluid simulations and inform ML models initially trained on a large number of simulation results (full simulation set). Simulation results alone showed that hemodynamics in patients with CoA were nonlinear and highly complex. Using our framework, we were able to identify the minimal simulation set needed to create a patient-specific prediction model capable of capturing the full spectrum of synergistic factor coupling. We then successfully tested our ML predictions against the full patient simulation set, demonstrating that the ML framework can accurately determine the impact of risk factors on key metrics for CoA. Finally, we tested the ML model in two new patient data sets and found that we could predict Δ*P* accurately (Fig. 1). Our findings lay the conceptual foundation for developing simulation driven ML models that are capable of predicting the impacts of a wide array of physiological factors on vascular diseases.

## Results

### Development of massively parallel models to simulate impacts of interacting factors

The first critical step to developing our framework was to perform blood flow simulations accounting for various physiological factors. Flow characteristics were simulated using HARVEY, a massively parallel hemodynamics application^15^. HARVEY is based on the lattice Boltzmann method (LBM) for solving the Navier-Stokes equations^30,31^. Details of the implementation of the LBM in HARVEY can be found in Methods. HARVEY is written in C/C++, parallelized with MPI/OpenMP, and demonstrates excellent parallel scalability for simulations in vascular geometries^13^. In this study, all simulations were run on the Blue Gene/Q Vulcan supercomputer at Lawrence Livermore National Laboratory (LLNL) with each simulation using between 1,024 and 4,096 nodes (16 cores per node). A 50 *μ*m resolution was needed to model the fluid and vessel geometry to obtain accurate simulations based on the results of a convergence test, resulting in over 240 million fluid points and 6 million wall points in the aorta. However, many of the simulations required resolutions as low as 20 *μ*m to improve stability, resulting in over 3.7 billion fluid points and 38 million wall points. These high resolutions allowed the simulations to capture complex flow patterns including turbulent flows without the need for an explicit turbulence model. While multi-resolution LBM schemes could reduce the number of fluid points, our simulator uses a constant resolution for simplicity. The influence of resolution on numerical stability is described in Methods.

**Figure 2:**
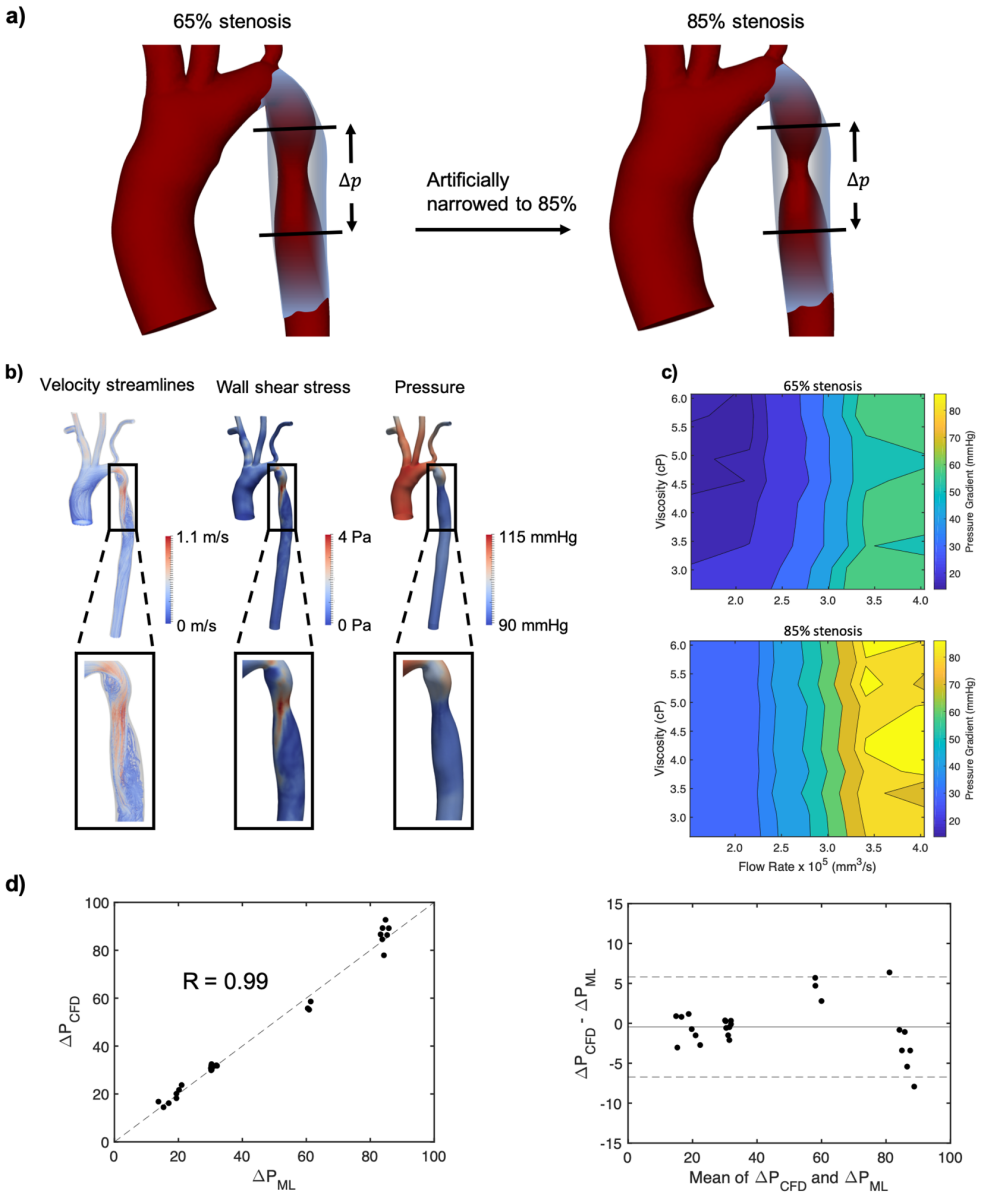
Computing Δ*P* with simulation and ML models. (**a**) The 65% and artificially narrowed 85% stenosis geometries (Blender version 2.77 - www.blender.org). (**b**) Sample velocity, WSS, and pressure outputs for the 65% stenosis geometry. (**c**) Contour plots showing how Δ*P* relates to viscosity and flow rate in the 65% and 85% stenosis geometries. (**d**) ML results comparing predicted and simulated Δ*P* with an associated Blandt-Altman plot.

Blood flow was simulated in CoA patients to capture a range of physiological factors, manifested in variations in viscosity and cardiac waveforms, to provide an accurate picture of the interplay between these parameters and how they influence pressure and TAWSS, allowing us to discern any potentially synergistic relationships. Five waveforms were simulated ranging from resting conditions to exercise (Fig. 1b). Simulations were performed in a patient-derived 65% (Fig. 1a) degree of stenosis (DoS) and an artificially narrowed 85% (Fig. 2a) DoS (details on the geometries can be found in Methods). Ten different blood viscosities spanning the physiological range (2.66–6.38 cP) were simulated for each flow waveform^32^, allowing simulations to capture a large range of viscosities including extreme conditions. Combining the five waveforms, ten viscosities, and two DoS values resulted in 100 simulations. Our parameter space included greater sample variations of viscosity and flow rates compared with DoS as we were interested in studying the impacts of potential future co-factors. An additional 36 simulations were performed in two more patient-derived geometries to test our ML framework.

Due to the high resolutions necessary for convergence and stability and the large geometries, over 70 million compute hours were used on the Vulcan supercomputer for all 136 blood flow simulations, the largest computational study of CoA to date. These simulations were a critical step to develop a high-fidelity mapping of how viscosity and flow rate impact complex hemodynamic metrics like Δ*P* and TAWSS, that require high-resolution 3D models to capture.

### Developing machine learning models for accurate pressure gradient predictions

Simulation results showing how viscosity and flow rate impact Δ*P* demonstrate the nonlinear complexity of hemodynamics in a CoA and the need for methods to predict results spanning a wide range of physiological factors. Sample simulation results for velocity, pressure, and TAWSS in the 65% CoA are shown in Fig. 2b. Δ*P* values in the 65% and 85% CoA were plotted against viscosity and flow rate for each DoS, as shown in the contour plots in Fig. 2c. The cross-sectional averaged peak to peak Δ*P*, which occurred at peak systole, was computed throughout this work, consistent with clinical CoA measurements^19^. In general, Δ*P* was larger across the 85% stenosis than across the 65% stenosis and increased with increasing flow rate for both geometries. Δ*P* increased with flow rate by up to four-fold and three-fold across the 65% and 85% stenoses, respectively. In the 65% stenosis, at flow rates below ~$2.5$ mm^3^/s, lower viscosity led to higher Δ*P*, while at flow rates greater than ~3 mm^3^/s, variations in viscosity produced sporadic changes in Δ*P*, likely due to turbulent conditions and disturbed flow patterns indicated by high Reynolds numbers (over 3,000).

These nonlinearities demonstrate that results cannot be predicted with simple linear ML models like ordinary least squares regression, and instead require nonlinear models. All ML models in this study were performed using neural networks to account for these nonlinearities. Details regarding the neural network models are described in Methods. To determine ML prediction strength, we used similar methods to^24^ and^26^ and computed the correlation coefficient between the predicted results with the ML model ($\Delta {P}_{{\rm{ML}}}$ or TAWSS_ML_) and the simulation results ($\Delta {P}_{{\rm{CFD}}}$ or TAWSS_CFD_) in the test set. We also performed Blandt-Altman analyses with every ML model to ensure minimal bias. Input features in this model were DoS, heart rate, peak flow rate at the aortic root, and viscosity. 70 simulations were used in the training set, 10 simulations were used in the validation set, and 20 simulations were used in the test set. 5-fold cross-validation was used in the training and validation sets to find the optimal model and ensure minimal overfitting. Figure 2d showed a strong correlation R = 0.99 between the simulation results and ML model predictions in the test set.

### Developing machine learning models for accurate TAWSS predictions

**Figure 3:**
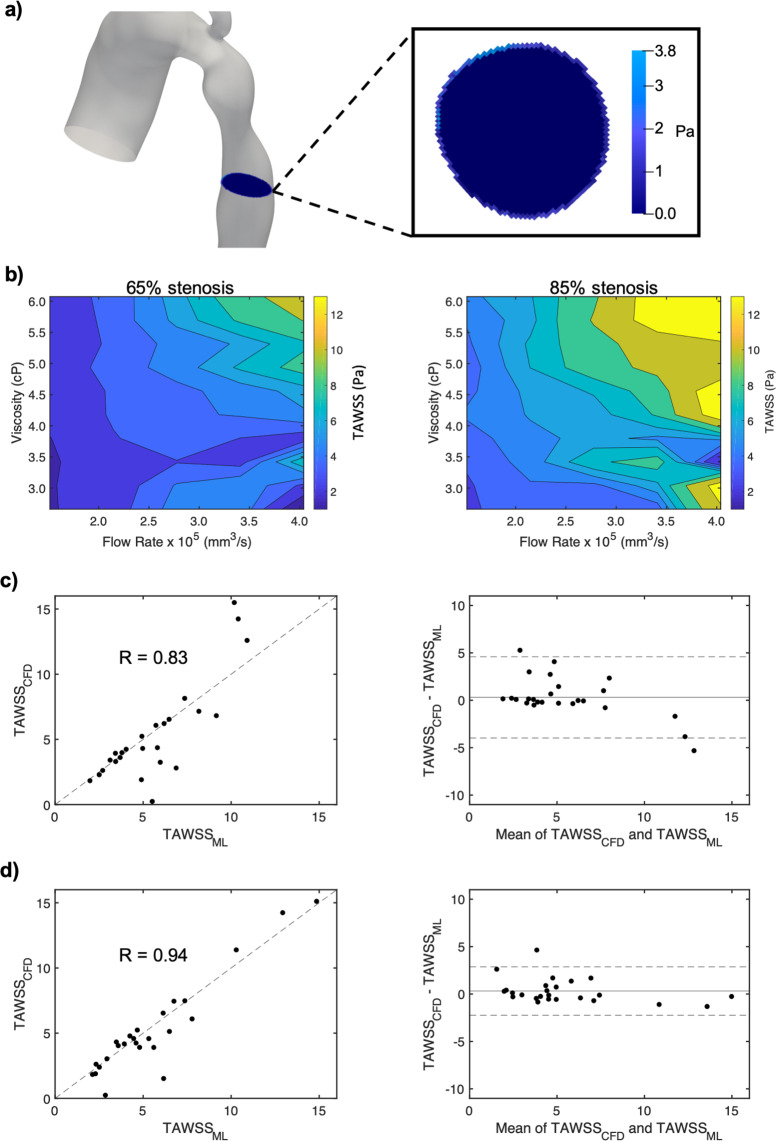
Computing TAWSS with simulation and ML models. (**a**) Circumferentially averaged TAWSS was computed in a transverse slice distally adjacent to the stenosis. (**b**) Contour plots showing how TAWSS relates to viscosity and flow rate in the 65% and 85% stenosis geometries. (**c**) ML results comparing predicted and simulated TAWSS with an associated Blandt-Altman plot. (**d**) ML results were extended to include WSS at three early time points during the cardiac cycle (0.035, 0.07, and 0.105 seconds) as additional input features with an associated Blandt-Altman plot.

To continue probing the capabilities of blood flow prediction models, we showed that simulation based ML models could also predict the results for TAWSS. TAWSS was calculated in a transverse slice distally adjacent to the CoA (Fig. 3a) (TAWSS calculations are described in Methods). Contour plots elucidating irregular and nonlinear relationships between circumferentially averaged TAWSS with viscosity and flow rate are shown in Fig. S1b. TAWSS was also computed by dividing the slice into four regions (Supplementary Fig. S1a). In both the 65% and 85% stenoses geometries, the highest TAWSS values were located in sectors one and two (Supplementary Fig. S1b). TAWSS increased with flow rate and viscosity for sectors one, two, and for the circumferential average, showing a relatively consistent pattern between both geometries (Fig. S1b and Supplementary Fig. S1c–f). In general, TAWSS was higher for most flow rates and viscosities in the 85% stenosis. Overall, the 65% and 85% stenoses demonstrated a positive but nonlinear and complex correlation between TAWSS with both viscosity and flow rate.

A neural network model was built with all 100 simulations in the 65% and 85% CoA using DoS, heart rate, peak flow rate at the aortic root, and viscosity as input features and TAWSS as the output. Once again, 70 simulations were used in the training set, 10 simulations were used in the validation set, and 20 simulations were used in the test set with 5-fold cross-validation. Simulation results for TAWSS were plotted against ML results. Interestingly, the prediction power decreases for TAWSS (R = 0.83, Fig. 3c) compared with Δ*P* (R = 0.99, Fig. 2d). To improve results for TAWSS, we tested the impacts of including WSS values as additional features at three time points towards the beginning of the cardiac cycle ($t=0.035,0.07$, and $0.105$ seconds). The additional features improved the correlation significantly to R = 0.94 (Fig. 3d), indicating that we were able to improve TAWSS predictions with much shorter simulations. The full cardiac cycles were 0.7 seconds for resting conditions and 0.42 seconds for exercise. Therefore, simulations could be performed in 15% of the total run-time for resting conditions and 25% for exercise conditions, a major advantage given the expensive computational demand of each individual simulation.

### Design of experiments to determine necessary simulations to accurately train machine learning models

Our main goal using priciples from design of experiments was to fully explore the impacts of viscosity and flow rate combinations on Δ*P* with reduced computational demand. To capture the entire parameter space, we trained and tested ML models capable of predicting Δ*P* using all 50 simulation results within the 65% CoA to determine a framework that works well for geometries at a given stenosis degree. Geometries with varying DoS raise the complexity of the problem and would likely require more features and simulations in the input set for accurate predictions. Instead of relying on the entire set of 50 simulations, we sought to find a reduced number of carefully chosen simulations that could maintain accurate predictions and test these results on two other CoA geometries. Input features for the ML model were viscosity, flow rate, and heart rate.

Here, we describe the design of experiments workflow depicted in Fig. 1c. The simulation results in the 65% CoA (Fig. 1c, step 1) were used to train and test the neural network models. The training and test sets initially consisted of 40 and 10 simulations, respectively. After developing the first neural network model, we successively reduced the number of simulations in the training set until the correlation coefficient dropped below R = 0.98, a threshold value based on initial correlation results using all 50 simulations (Supplementary Fig. S2). To remove a sample from the training set, the simulations comprising the reduced training set were randomly selected 1,000 times, and the training set that produced the best model was used. For example, if the training set consisted of nine simulations, 1,000 random samples of nine simulations were used to determine which set produced the best results. When the first simulation was removed (from 40 to 39 simulations resulting in 40 possible combinations of training sets), all combinations were tested. For each reduction in the training set size, the remaining simulations were used in the test set. For example, if the training set contained 20 simulations, the test set included all 30 remaining simulations. This increase in test set size allowed us to better span the outcome variable space. We note that each simulation can be defined by its viscosity-flow rate pairing (Fig. 1c, steps 2 and 3). After implementing the algorithm through Fig. 1c, step 3, we found that only nine simulations in the training set were needed to predict Δ*P* across the entire parameter space with R >= 0.98, a massive reduction in the number of necessary simulations. The nine viscosity-flow pairings for the 65% stenosis geometry are listed in Supplementary Table S1. We further expanded the framework to test on the 85% stenosis geometry, which required a different minimal simulation set of 21 viscosity-flow rate pairings for accurate Δ*P* predictions (Supplementary Table S2). This significantly larger minimum simulation set was likely due to increased flow complexity downstream of the 85% stenosis and higher turbulence within the stenosis.

Finally, we tested how this minimum simulation set performed on two additional patient-derived CoA geometries to show that the minimal simulation set in the 65% stenosis was not specific to a single geometry but could be used for a variety of geometries with a 65% stenosis. For each new geometry, the training set was developed by performing simulations with the same nine viscosity-flow rate pairings from the minimum simulation set of the original CoA. Nine more simulations consisting of a random selection of viscosity-flow rate pairings were used to test the model (Fig. 1c, step 4). Thus 18 simulations were required for each of the two new geometries, leading to a total of 36 additional simulations. Neural networks were built from the simulations in each new geometry, and correlation coefficients were computed in the test sets (Fig. 1c, step 5). The results for the two new CoA geometries are shown in Fig. 1d,e, and both correlation coefficients were R = 0.98, demonstrating accurate predictions with ML models built from the minimal simulation set. These results demonstrate that the ML model could accurately predict Δ*P* in other geometries with the same DoS with only a small number of additional simulations.

### Validation of blood flow simulation methods under unsteady conditions

**Figure 4:**
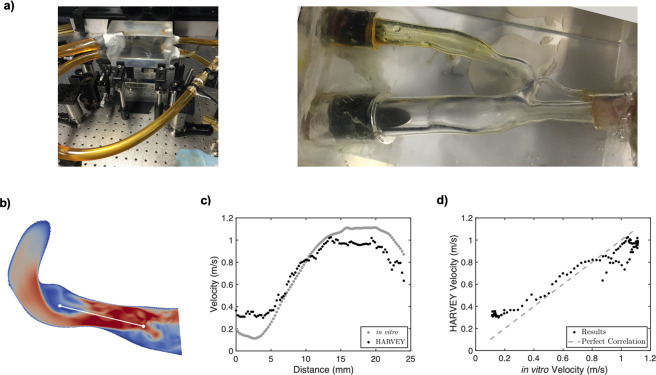
Validation of blood flow simulation methods. (**a**) PIV setup. (**b**) Results from HARVEY were compared with *in vitro* flow by plotting time-averaged velocity along a line through the center of the CoA. (**c**,**d**) HARVEY comparisons with *in vitro* flow.

To ensure that the data being used for training and test sets is accurate, it was critical to first ensure the accuracy of the software and the simulation set-up through comparison with *in vitro* flow measurements. To validate the simulation, calculated velocity profiles are compared to those measured in a controlled flow experiment measured in a 3D-printed vasculature from the same mesh input file used in the simulation. Such an *in vitro* result is useful as the geometries can be constructed with precise dimensions, and flow properties such as velocity and pressure can be easily measured^33,34^. In this case, the CoA geometry with a 65% stenosis was used. HARVEY has been previously validated under steady flow conditions by comparing simulated flow to *in vitro* results (RMSD = 0.042) in this 3D-printed geometry using particle image velocimetry (PIV)^35^. The experimental setup for our study is described in Methods and a picture of the setup is shown in Fig. 4a. The PIV experiment was modified for pulsatile flow with an inflow waveform that exactly matches the patient-specific resting waveform shown in Fig. 1b with a cardiac cycle time of 0.7 seconds. Data were acquired for reference times between 100–600 ms at 11 evenly spaced time points separated by intervals of 50 ms. Comparisons were performed by computing velocity along a line through the center of the CoA (Fig. 4b). The velocity profiles were averaged over every time point and plotted in Fig. 4c. Time-averaged differences between simulated and *in vitro* results (RMSD = 0.137) are shown in Fig. 4d. These results further validate HARVEY by demonstrating the similarity between computationally and experimentally simulated pulsatile flow. Additional validation of our methods was performed by comparing simulated and *in vivo* measurements of Δ*P* across a 65% stenosis for a patient at rest with normal blood viscosity. Under these conditions, the simulated and *in vivo* Δ*P* were 17.8 and 17.3 mmHg, respectively^36^.

## Discussion

Our results demonstrate the strong potential of a combined ML/design of experiments framework for overcoming the computational burden associated with many physics-based simulations. It is often critical to explore a massive parametric space to obtain a robust clinical profile and understand how physiological parameters influence disease severity. However, the large number of parameter combinations alongside the prohibitive computational demand of an individual simulation can make such studies impractical on a per-patient basis. Individual blood flow simulations are highly computationally demanding due to complex image-derived geometries, pulsatile flow rates, and realistic outlet boundary conditions. To overcome this challenge, we present a framework that uses principles from design of experiments to produce accurate ML models with a limited number of blood flow simulations. While others have shown that ML can be used to fully explore parameter spaces in various fields of study such as photovoltaics^37^, our framework offers the ability to develop ML models capable of exploring future risk on a per-patient basis. With over 1,600 babies born with CoA and approximately 40,000 born with congenital heart defects each year in the U.S. alone^17,38^, models that are adaptable to individual patients have incredible potential to guide treatment planning and improve patient outcomes.

A key innovation in this work is the use of design of experiments principles to drive the use of neural network models. This combination enabled clinical outcome metrics to be produced in less than one second. By building our ML models from 3D simulations, we ensure our predictions arise from high-fidelity data and rely on our physics-based model to create sufficient training and testing data. While other approaches have been proposed to address the high computational cost of 3D blood flow simulations, including 1D and lumped parameter models that significantly reduce the degrees of freedom of the model and decrease the computational cost, these rely on limiting assumptions reducing the scope of the results. Research by^39^ and^40^ indicated that simulating a cardiac cycle with 1D models can take on the order of minutes, whereas lumped parameter models can take on the order of seconds. However, the key difference is that 1D and lumped parameter models cannot capture local variations in hemodynamic profiles.

Our initial step towards this goal was to determine the general characteristics of the relationships between viscosity and flow rate with Δ*P* and TAWSS in patients with CoA. To accomplish this task, we simulated 50 viscosity-flow rate pairs in two CoA geometries (85% and 65% stenoses), requiring over 70 million compute hours. These results followed similar trends observed by^1^ who showed markedly increased Δ*P* and TAWSS in exercising patients and patients with narrower stenoses. However, by simulating 100 cases, we were able to capture local variations in flow that have not been shown in previous work. These complex relationships demonstrate the difficulty in accurately predicting clinically relevant parameters in physiological flow conditions. Additionally, they show the need for 3D geometries to capture flow complexities that may be lost with simpler models.

By combining physics-based simulation with ML, we introduced a generalizable approach that can be used to predict key hemodynamic metrics on a per-patient basis, in minimal time, and relying on a reduced computational load. We chose to use neural networks for our ML models due to the nonlinearities observed from 3D simulations and from the excellent results shown by^24,26^, and^25^, who showed that neural networks can replicate the results of blood flow simulations. The ML model using all 50 simulations for the 65% stenosis geometry showed powerful prediction results when 40 simulations were used in the training set. However, the importance of minimizing the number of simulations becomes clear considering that these 40 simulations required over 20 million compute hours, all for a single patient. To build accurate ML models tuned per-patient, we needed to develop a technique to drastically reduce the required number of simulations. Using the methodology shown in Fig. 1, we found that only nine simulations were required to train the model with a high degree of accuracy (R = 0.98), reducing the compute time by a factor of four. Training and testing each ML model took less than one second and was therefore negligible in terms of total run-time. These nine viscosity-flow rate pairings also produced excellent ML models in two other patients with 65% CoA (R = 0.98 for each patient). This crucial component of the study shows the robustness of our framework and potential for wide applicability to new patients.

We focus this work on patients with CoA, but our approach could be applied to predict risk for patients suffering other vascular diseases. While various diseases could require the inclusion of other parameters as inputs to the model, such as ventricular pressure for predicting the functional severity of coronary lesions or larger vascular geometries to study peripheral arterial disease, the general framework (Fig. 1) is extendable to these models. Additionally, this work examines two of the most studied factors for CoA (Δ*P* and TAWSS), but the framework developed and presented here could be used to investigate other metrics of interest like oscillatory shear index and vorticity, which have both been shown to impact plaque progression in arteries^41,42^.

Predicting the effects of physiological factors on hemodynamics in patients with congenital heart disease can improve treatment plans and long-term outcomes. In this study, we developed 3D simulation-based ML models to predict how physiological factors impact clinically relevant diagnostic hemodynamic parameters in patients with CoA. Capturing all possible combinations of factors with simulations is intractable, but using principles from design of experiments to pick the minimal set of simulations that yield accurate ML models can greatly improve the diagnostic potential of these predictions. Our framework provides a foundation for physicians and researchers to accurately and quantitatively predict the impacts of physiological factors on patient hemodynamics before they occur.

## Methods

### Assumptions in blood flow simulations

To simplify the experimental design, vessel walls were assumed to be rigid. This assumption has been commonly used in previous well-validated CoA simulation models^1,43^, and several studies have shown only minor impacts to flow when incorporating vessel wall elasticity^44–46^. However, including deformable walls better represents realistic vascular geometries and can be included for comparison in future work. Additionally, blood was modeled as a Newtonian fluid, which is generally a valid assumption in large blood vessels where shear rates are greater than 100 *s*^−1^ ^47^. Parabolic flow profiles derived from inlet waveforms obtained from the Vascular Model Repository (www.vascularmodel.com) were imposed at the base of the ascending aorta using the finite difference boundary condition^48^. The computed Δ*P* locations near the stenosis were assumed to be valid. These locations were chosen based on the 2012 MICCAI CFD Challenge established as a part of the medical imaging conference as a means to provide consistency, repeatability, and most importantly accuracy as compared to invasive pressure measurements^36^. Additionally, our results at these locations match the *in vivo* Δ*P*. While we showed that our framework can be used to predict Δ*P* with minimal simulations in CoA geometries with the same DoS, other DoS’s will require different simulations. Therefore, future work can include a higher number of DoS’s in the models.

### Implementing the lattice Boltzmann method in Harvey

The LBM divides the geometry into a fixed Cartesian grid. At each grid node, the fluid is represented by a probability distribution function denoted ${f}_{i}(x,t)$, indicating the likelihood that a particle is located at grid point $x$, at time $T$, and traveling with discrete velocity ${c}_{i}$. The fluid dynamics are resolved through the time evolution of ${f}_{i}(x,t)$ by the LBM equation:

1 $${f}_{i}(x+{c}_{i}\Delta t,t+\Delta t)-{f}_{i}(x,t)=-\,\Delta t\Omega ({f}_{i}(x,t)-{f}_{i}^{eq}(x,t))$$

HARVEY uses a standard D3Q19 lattice with a single relaxation time Bhatnager-Gross-Krook (BGK) collision operator.

There are two key components of the algorithm: advection and collision. In the advection portion of each time step, particles are propagated along the velocity paths to their neighboring nodes defined by the lattice discretization. Fluid-fluid collisions are then handled through a relaxation towards a local equilibrium, shown on the right-hand side of Eq. 1. HARVEY uses a half-way bounce back boundary condition at the vessel walls and a finite difference boundary condition at the inlets and outlets^49^.

Lattice Boltzmann pressure (${P}_{LB}$) and density ($\rho$) are related through the ideal gas equation:

2 $${P}_{LB}={c}_{s}^{2}\rho $$

where ${c}_{s}$ is the lattice speed of sound. ${P}_{LB}$ is denoted here with a subscript to differentiate the value from physical pressure ${P}_{PHYS}$. It should be noted that values are non-dimensionalized prior to the LBM simulation. Each value is non-dimensionalized with a characteristic time ($\delta t$), a characteristic length ($\delta x$), and the physical fluid density. After completion of the simulation, these values are then re-dimensionalized into physical units for analysis. For example, multiplying *u* by $\delta x/\delta t$ converts the non-dimensional velocity into units of m/s. However, re-dimensionalizing ${P}_{LB}$ is not as straightforward and is described in methods.

The stability of the simulation is influenced by the Mach number (Ma):

3 $${\rm{Ma}}=\frac{{u}_{0}}{{c}_{s}}$$

where *u*_0_ is the peak velocity of blood at the inlet. Raising the resolution increases the stability of the LBM simulation by reducing the Ma. The Ma introduces compressibility error into the simulation and was held as small as possible (Ma $\ll$ 1)^50^. The time step was chosen based on the physiological blood viscosity ${\nu }_{PHYS}$ and the prescribed non-dimensional lattice Boltzmann viscosity ${\nu }_{LB}$:

4 $$\delta t=\delta {x}^{2}\frac{{\nu }_{LB}}{{\nu }_{PHYS}}$$

### Outlet boundary conditions to account for downstream resistance

A resistance model was imposed at the outlets of the geometries^51–53^:

5 $${P}_{i}={Q}_{i}{R}_{i}$$

*P*_*i*_, *Q*_*i*_, and *R*_*i*_ are the pressure, flow rate, and downstream vascular resistance at the i^th^ outlet, respectively. ${R}_{i}$ was tuned iteratively at each outlet so that the simulated and *in vivo* flow rate matched. For example, increasing the resistance at the dAo outlet would raise the pressure at that outlet, thereby reducing the flow rate through the dAo. Flow rates through each outlet were assumed to be constant across all waveforms in the 65% stenosis. However, in the 85% stenosis, flow rates through the dAo were expected to decrease due to the narrower stenosis. This was naturally accounted for by matching resistance values in the 85% stenosis to the 65% stenosis simulations that used the same flow waveform.

*R*_*i*_ values were inputted into HARVEY with units of Pa · s/m^3^ and non-dimensionalized using the density, $\delta t$, and $\delta x$. ${Q}_{i}$ was computed from the outward normal velocities at each grid point in the i^th^ outlet. From Eq. 5, $\rho$ was substituted for pressure as the LBM computes $\rho$ rather than pressure at each time step. The equation implemented in HARVEY is shown here:

6 $${\rho }_{i}={Q}_{i}{R}_{i}+1$$

A constant of one was added to the right-hand side of Eq. 6 because a $\rho$ value of one typically corresponds to zero pressure.

### The relationship between lattice Boltzmann pressure and physical pressure

As with Navier-Stokes solvers, an unknown constant is required to convert lattice Boltzmann pressure ${P}_{LB}$ to a manometric unit such as mmHg^54^. We determined this constant by scaling the ${P}_{LB}(t)$ at the base of the aorta to fit a physiological pressure waveform ${P}_{PHYS}(t)$ in mmHg. We selected two physiological pressure waveforms from the Vascular Model Repository^55^, appropriate for the rest and exercise states. In the case of the rest state, *in vivo* data at the base of the aorta were available for systole and diastole: 115 and 65 mmHg respectively. The pressure waveform was adjusted to match these values.

For each simulation, a linear equation relating ${P}_{LB}(t)$ and ${P}_{PHYS}(t)$ was derived using least squares regression, using data at 20 evenly spaced time points in the cardiac cycle. The resulting conversion could be used to compute ${P}_{PHYS}$ throughout the geometry, at any point in the cardiac cycle. In particular, we computed ${P}_{PHYS}$ above and below the CoA from the simulated ${P}_{LB}$ to approximate the pressure gradient.

### Calculating time-averaged wall shear stress

As many studies have shown correlations between TAWSS and plaque progression in the dAo, we computed TAWSS at a transverse slice distally adjacent to the stenosis^21–23^. The slice was divided into four sectors, and the mean TAWSS in each sector was compared (Supplementary Fig. S1a,b). Analyzing TAWSS in sectors provides valuable information regarding TAWSS heterogeneity. Additionally, previous studies have shown statistically different results between circumferentially averaged TAWSS and TAWSS divided into sectors^35,56^. The circumferential average as well as the sectors with the highest mean TAWSS were used for further analysis (Fig. 3 and Supplementary Fig. S1c–f). Sectors with high TAWSS were chosen because we observed that TAWSS was greater in narrower stenoses, potentially indicating that a more severe CoA results in higher TAWSS in the dAo. TAWSS was calculated acccording to:

7 $${\rm{TAWSS}}=\frac{1}{T}\,{\int }_{0}^{T}\,|{\rm{WSS}}|dt,$$

where $T$ is the simulation time for one cardiac cycle, and WSS is the wall shear stress. Computing WSS in the LBM is described in detail in^57^.

### Patient-derived geometries and flow rates

3D geometries are important in arterial blood flow simulations for capturing complex vasculature. In idealized conditions such as fully developed flow in a pipe, the change in flow parameters is expected to follow a simple and predictable relationship with flow rate and viscosity. In contrast, realistic blood vessel geometries and patient-derived pulsatile waveforms and boundary conditions lead to highly intricate flow patterns, particularly in geometries with a stenosis. 3D simulations are necessary to fully capture the flow complexities and produce accurate results. This need has been confirmed by^1^ who showed that Δ*P* and TAWSS in an image-derived geometry were complex, and that the relationship between flow rate and Δ*P* depended on the specific geometry. Others have used lumped parameter and 1D models derived from 3D geometries to model blood flow. Although the run-time and memory requirements are significantly reduced, these models make limiting assumptions, increase uncertainty, and lose local and detailed information such as WSS that is important in understanding patient hemodynamics^27,28^.

Blood flow was simulated in 3D geometries derived from patient data. The patient-specific aortic geometry was provided by the Vascular Model Repository (www.vascularmodel.com). The patient was an 8-year-old female with a 65% stenosis. The aorta was imaged using gadolinium-enhanced magnetic resonance angiography (MRA) with a 1.5-T GE Signa scanner. A segmented mesh file was created using customized SimVascular software^58^ and was provided in stereolithography (STL) format^36^ (Fig. 1a). The second geometry analyzed in this study was created artificially by increasing the DoS to 85% using the graphics software Blender (Blender Foundation) (Fig. 2a). An 85% DoS was chosen to investigate how a narrow stenosis impacts hemodynamic factors^59^. Two additional image-derived geometries with artificially introduced 65% stenoses were used for the design of experiments component of this study (Fig. 1c step 4). These two geometries, also obtained from the Vascular Model Repository (www.vascularmodel.com), were from 3- and 11-year-old females respectively.

Pulsatile flow rates were measured during rest at the base of the aorta for the duration of a cardiac cycle by phase contrast magnetic resonance imaging (PC-MRI) sequence encoding^36^. Two additional rest waveforms were derived by scaling the patient-specific waveform by constant factors. The exercise (stress) waveform was derived from a different patient in the Vascular Model Repository, also with a CoA, whose heart rate was raised via a pharmacological stress test, as no stress waveform was measured for the patient-specific geometry used in this study. Because this patient was an 18-year-old male, the waveform was scaled to appropriate physiological values^55^. Two different constants were used to scale the stress waveforms to provide a range of inlet flow rates. The scaling constants were chosen so that the peak flow rates in each waveform were evenly spaced between the smallest and largest waveforms (1.51*e*5–4.03*e*5 mm^3^/s) while retaining the same qualitative shape^1^. We note that heart rate and flow rate are variable among patients, and different activities will influence these parameters. These ranges were chosen as a representative example to demonstrate our ML framework. The pulsatile waveforms are shown in Fig. 1b. All simulations used pulsatile flow, and simulations were performed for the duration of one cardiac cycle.

### Neural network model design

Based on simulation results (Figs. 2b,c and 3b), the relationship between viscosity and flow rate with the output metrics Δ*P* and TAWSS was nonlinear and complex. Therefore, a simple linear model was unlikely to suffice, and we decided to account for increased complexity with a neural network model. The neural network contained a single hidden layer consisting of ten nodes (Fig. 1). We tested more nodes in the hidden layer as well as an increased number of hidden layers and found no significant difference in predictive outcomes. Additionally, we tested other nonlinear ML models including regression trees and support vector machines and found slightly worse prediction results. All neural network models were trained and tested using TensorFlow^60^ with the application programming interface (API) Keras (https://keras.io/). The models were fully connected networks with rectified linear unit (ReLu) activation functions at the hidden layer and a linear activation function at the output layer. The ReLu activation function within the hidden layer has been shown to fit experimental data well and is commonly used in neural network models^61^. The models were optimized using the adaptive moment estimation (ADAM) optimization algorithm and a mean squared error loss function. The ADAM optimizer is also a popular choice due to its superior performance compared with several other gradient descent algorithms^62,63^. To prevent overfitting, early stopping was used when the mean squared error no longer decreased. Input data was scaled prior to model development to mean zero and standard variation one. Several different neural network models were created depending on the studies performed described in Results. The same architecture was implemented for each model, but the input features and outputs varied. The models are briefly summarized in Supplementary Table S3.

### Validating blood flow simulations with *in vitro* methods

The *in vitro* studies were performed in a patient-specific physical, lost-core urethane model of the 65% CoA embedded in a mechanical flow loop^33^. Pulsatile flow was driven with a custom computer-controlled piston pump^64^. The inlet and outlets of the CoA were connected to the flow loop with flexible Tygon tubing (R-3603, Ryan Herco Flow Solutions, Burbank, CA). The transition region from the pump to the tubing was milled so that it was optimally smooth, and the model was then configured such that the tubing was flush to the inlet. A sodium-iodide based solution with a viscosity of 2.05 cP and density of 1,750 kg/m^3^ was used to mimic blood. The fluid was carefully chosen such that its refractive index matched the urethane CoA model (*n* = 1.49) to eliminate any optical distortion during PIV. The PIV system consisted of a LaVision 3D Flowmaster (LaVision, Ypsilanti, MI, USA) with two $1,476\times 1,040$ pixel Imager Intense PIV CCD cameras with 6.45 *μ*m square pixels. Each camera contained AF Micro Nikkor 60 mm lenses (Nikon, Tokyo, Japan) with lens *f* numbers of 8 and was placed approximately 55 tube diameter lengths downstream of the inlet in a stereographic configuration centered for optimal imaging. PIV was used to capture velocity fields in a vertical plane passing through the center of the geometry. A 532 nm Gemini PIV dual cavity double-pulsed Nd:Yag laser (New Wave Research, Fremont, CA, USA) and various optics were used to form a double pulsed, 0.5 mm thick, vertical light sheet focused at the centerline of the stenosis. Silver coated hollow glass spheres (TSI Incorporated, Shoreview, MN, USA) having a mean diameter between 8–12 *μ*m and a density of 1.65 g/mL were used to scatter light. Low-noise measurements of particle displacement and velocity were obtained using standard stereo PIV procedures in two frame cross-correlation mode^65^. Images of the 3D CoA model embedded in the PIV system are shown in Fig. 4a.

The piston pump was setup to drive pulsatile flow with an inflow waveform that matched the patient-specific resting waveform shown in Fig. 1b with a cardiac cycle time of 0.7 seconds. Data were acquired at 11 evenly spaced time points separated by 50 ms intervals for the duration of the cardiac cycle.

## Supplementary information

Supplementary Information.

Supplementary Information2.
