# Supplementary material for: Accelerating massively parallel hemodynamic models of coarctation of the aorta using neural networks
Source: Sci Rep. 2020 Jun 11;10:9508. doi: 10.1038/s41598-020-66225-0 (PMC7289812; doi:10.1038/s41598-020-66225-0)
Supplement: Supplementary file 1 — Supplementary Information. [file 41598_2020_66225_MOESM1_ESM.pdf]

Accelerating massively parallel hemodynamic models of coarctation of the aorta using neural networks

Bradley Feiger[1], John Gounley[1], Daletnature Adler[3], Jane A. Leopold[3], Erik W. Draeger[2], Rafeed Chaudhury[4], Justin Ryan[4], Girish Pathangey[4], Kevin Winarta[4], David Frakes[4], Franziska Michor[5], and Amanda Randles[1, *]

[1]Department of Biomedical Engineering, Duke University, Durham, NC, USA

[2]Lawrence Livermore National Laboratory, Livermore, CA, USA

[3]Brigham and Women's Hospital, Harvard Medical School, Boston, MA, USA

[4]Department of Biological aond Health Systems Engineering, Arizona State University, Tempe, AZ, USA

[5]Department of Biostatistics and Computational Biology and Center for Cancer Evolution, Dana-Farber Cancer Institute, Boston, MA, USA; Department of Biostatistics, Harvard T. H. Chan School of Public Health, Boston, MA, USA; Department of Stem Cell and Regenerative Biology, Harvard University, Cambridge, MA, USA; The Broad Institute of Harvard and MIT, Cambridge, MA, USA.

[*]amanda.randles@duke.edu

Supplementary tables and figures

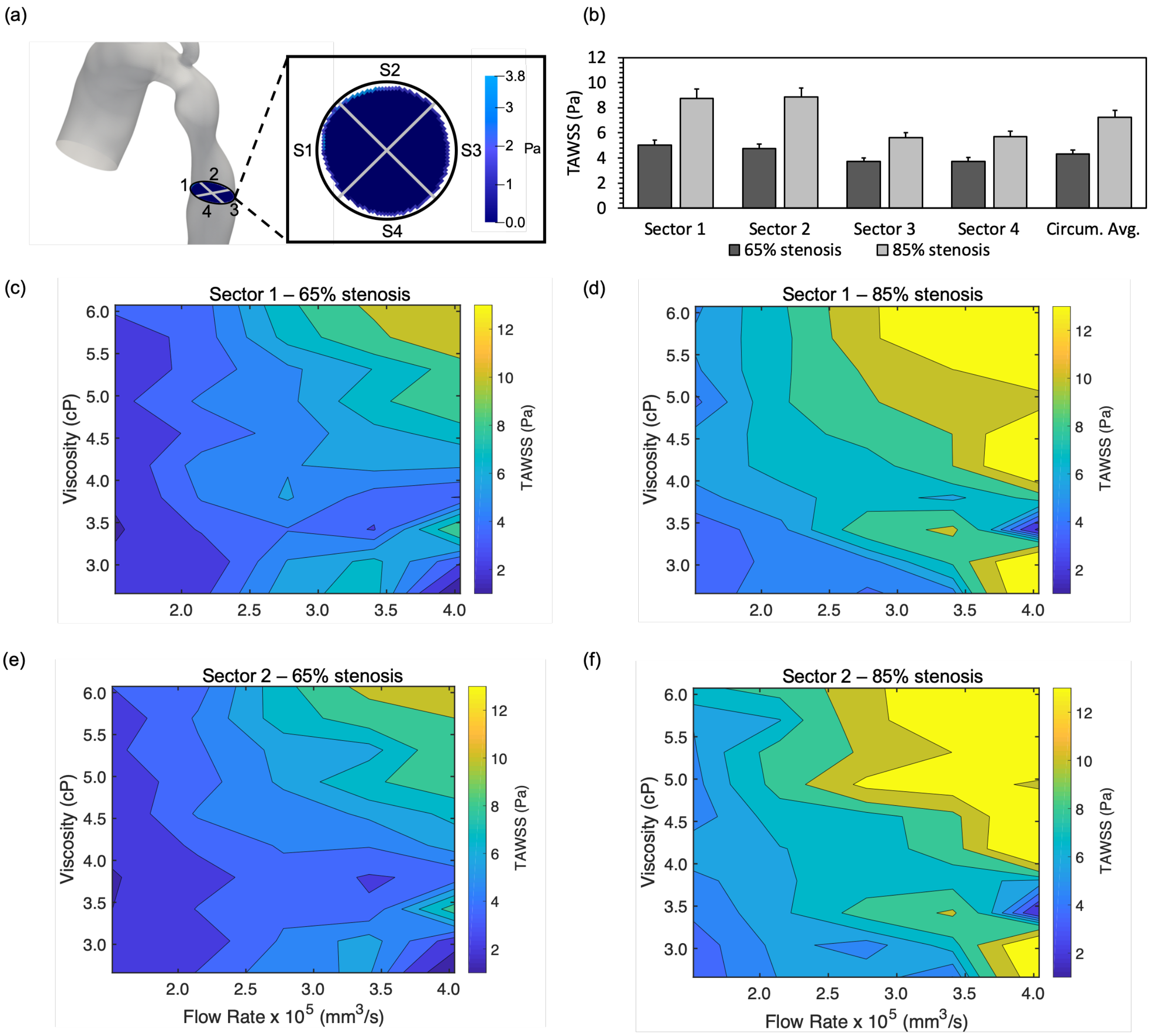

Figure S1: **Variation of TAWSS divided into sectors with viscosity, flow rate, and DoS. a,** TAWSS was divided into four sectors in a transverse slice distally adjacent to the CoA. **b,** The mean TAWSS in each sector as well as the circumferential average were compared. **c,d** TAWSS in sector one was computed in the 65% and 85% stenoses. **e,f** TAWSS was also computed in sector two in the 65% and 85% stenoses.

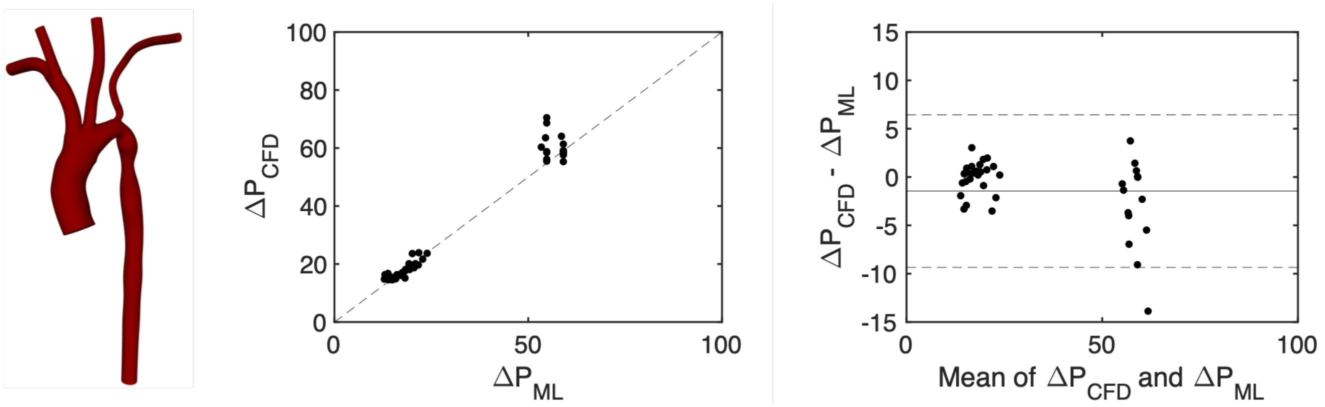

Figure S2: **ML results from the minimal simulation set are shown for the 65% stenosis geometry.** The training set consisted nine simulations (minimal simulation set), and the test set contained the remaining 41 simulations. A Blandt-Altman analysis was performed to show minimal bias.

Waveform	Peak Inlet Flow Rate (mm^3/s)	Viscosity (cP)
0.75*rest	151481.4	5.02
rest	201975.2	4.30
1.25*rest	252469.0	5.02
1.25*rest	252469.0	4.30
1.25*rest	252469.0	2.87
0.4*stress	323442.8	2.51
0.5*stress	403950.4	5.73
0.5*stress	403950.4	5.37
0.5*stress	403950.4	3.23

Table S1: The nine viscosity-flow rate pairings that, when simulated, could accurately predict ΔP in the 65% original geometry and two additional CoA geometries used for the design of experiments component.

**Waveform**	**Peak Inlet Flow Rate (mm^3/s)**	**Viscosity (cP)**
0.75*rest	151481.4	5.02
0.75*rest	151481.4	4.3
0.75*rest	151481.4	3.58
0.75*rest	151481.4	3.23
0.75*rest	151481.4	2.51
rest	201975.2	5.37
rest	201975.2	5.02
rest	201975.2	4.3
1.25*rest	252469	5.02
1.25*rest	252469	2.87
0.4*stress	323442.8	5.02
0.4*stress	323442.8	4.66
0.4*stress	323442.8	3.94
0.4*stress	323442.8	3.58
0.5*stress	403950.4	5.37
0.5*stress	403950.4	4.66
0.5*stress	403950.4	4.3
0.5*stress	403950.4	3.94
0.5*stress	403950.4	3.58
0.5*stress	403950.4	2.87
0.5*stress	403950.4	2.51

Table S2: The 21 viscosity-flow rate pairings that, when simulated, could accurately predict ΔP in the 85% geometry.

Parameter Predicted	Goal	Input Features	Figure
ΔP	Design of experiments to find the minimal simulation set needed to train accurate ML models in the 65% CoA geometry	Viscosity, flow rate, heart rate	Fig. 1, Fig. S2
ΔP	Develop ML models capable of predicting ΔP in 65% and 85% CoA geometries	Viscosity, flow rate, DoS, heart rate	Fig. 2
TAWSS	Develop ML models capable of predicting TAWSS in 65% and 85% CoA geometries	Viscosity, flow rate, DoS, heart rate	Fig. 3
TAWSS	Develop ML models capable of predicting TAWSS gradients in 65% and 85% CoA geometries with additional input parameters	Viscosity, flow rate, DoS, heart rate, WSS at three early timepoints in the cardiac cycle	Fig. 3

Table S3: A summary of the various ML models.
