# Supplementary figures and images for: Accelerating massively parallel hemodynamic models of coarctation of the aorta using neural networks
Source: Sci Rep. 2020 Jun 11;10:9508. doi: 10.1038/s41598-020-66225-0 (PMC7289812; doi:10.1038/s41598-020-66225-0)

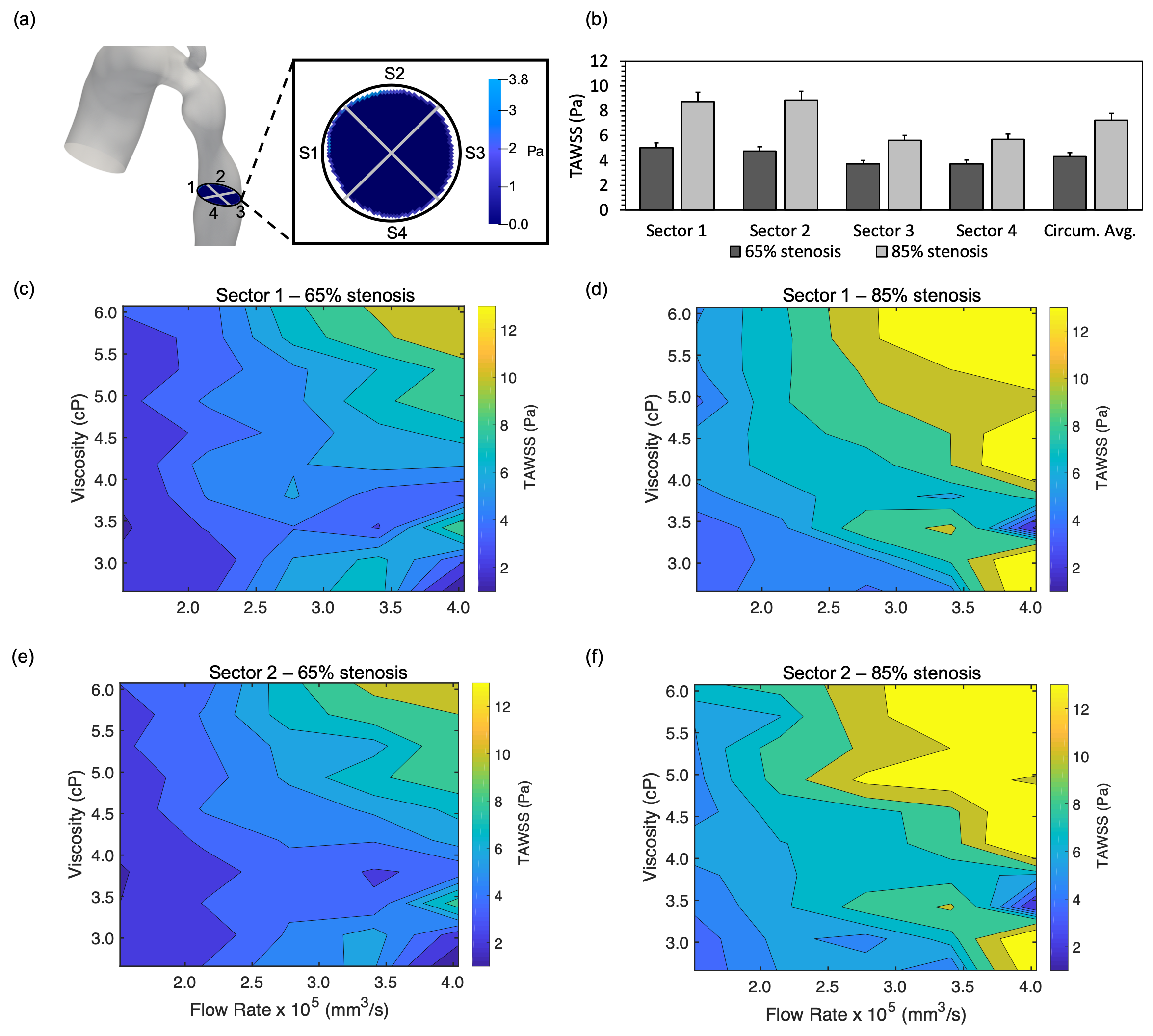

Supplement: Supplementary file 2 — Supplementary Information2. [file 41598_2020_66225_MOESM2_ESM.zip › tawss_suppl.png]

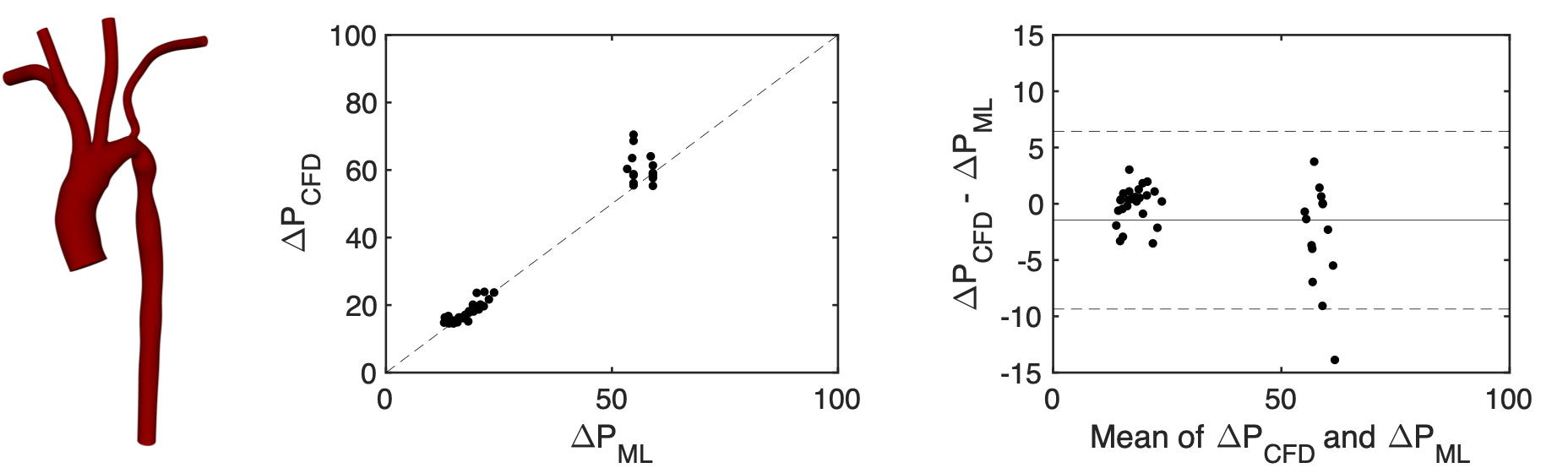

Supplement: Supplementary file 2 — Supplementary Information2. [file 41598_2020_66225_MOESM2_ESM.zip › original_geom_suppl.png]
